# Water nutrient concentration and temperature control microbial-driven decomposition of leaf litter in oceanic island streams

**DOI:** 10.17912/micropub.biology.002314

**Published:** 2026-09-02

**Authors:** Verónica Ferreira, Pedro M. Raposeiro, Vítor Gonçalves

**Affiliations:** 1 MARE – Marine and Environmental Sciences Centre, ARNET – Aquatic Research Network, Department of Life Sciences, University of Coimbra, Coimbra, Portugal; 2 University of the Azores, Faculty of Sciences and Technology, Ponta Delgada, Portugal; 3 CIBIO – Research Centre in Biodiversity and Genetic Resources, InBIO Associate Laboratory, BIOPOLIS Program in Genomics, Biodiversity and Land Planning – UNESCO Chair – Land Within Sea: Biodiversity & Sustainability in Atlantic Islands, University of the Azores, Ponta Delgada, Portugal

## Abstract

Litter decomposition fuels food webs in forest streams. Thus, understanding the effects of water nutrients and temperature on litter decomposition and decomposers is key for predicting stream functioning under environmental change. We incubated leaf litter of woody species in streams to assess the effects of nutrient concentrations and temperature on microbial-driven litter decomposition and microbial activities. The relationship between biotic and abiotic variables was generally best described by a quadratic model: there was a hump-shaped relationship between biotic variables and nitrate concentrations and a U-shaped relationship between biotic variables and temperature. The stimulatory effect of high nutrient availability was limited by low temperature, while high nutrient availability alleviated low-temperature effects on litter decomposition and decomposers.

**Figure 1. Relationships between biotic variables associated with leaf litter decomposition of three woody species and water nitrate concentration and temperature in six Azorean streams:** Figure 1. Relationships between biotic variables [microbial-driven litter decomposition rates (A), fungal biomass (B), and cumulative conidial production (C)] associated with leaf litter of three woody species ( *Acacia melanoxylon* , *Clethra arborea* , and *Pittosporum undulatum* ) and water nitrate concentration (left column) and temperature (right column) in six Azorean streams during Spring/Summer 2014. Stream labels: Lom1 – Lom4, streams located in Lombadas valley; AFG1 and AFG2, tributaries of Fogo lagoon. Table 1. Relationships between biotic variables (microbial-driven litter decomposition rates, fungal biomass, and cumulative conidial production) associated with leaf litter of three woody species ( *Acacia melanoxylon* , *Clethra arborea* , and *Pittosporum undulatum* ) and water nitrate concentration and temperature in six Azorean streams streams during Spring/Summer 2014. In most cases, data were fitted to a quadratic function: y = ax ^2^ + bx + c, where y is the biotic variable, x is the abiotic variable and a, b, and c are constants; a > 0 indicates a U-shaped curve and a < 0 indicates a hump-shaped curve. The curve vertex (i.e. the minimum value in a U-shaped curve or the maximum value in a hump-shaped curve) was calculated as: x = – (b / 2a) and y = ax ^2^ + bx + c. The coefficient of determination of the models (R ^2^ ) is also shown. The relationship between decomposition rates and nitrate concentration for *C.* *arborea* was fitted to a linear model (y = 0.00001x + 0.0078).

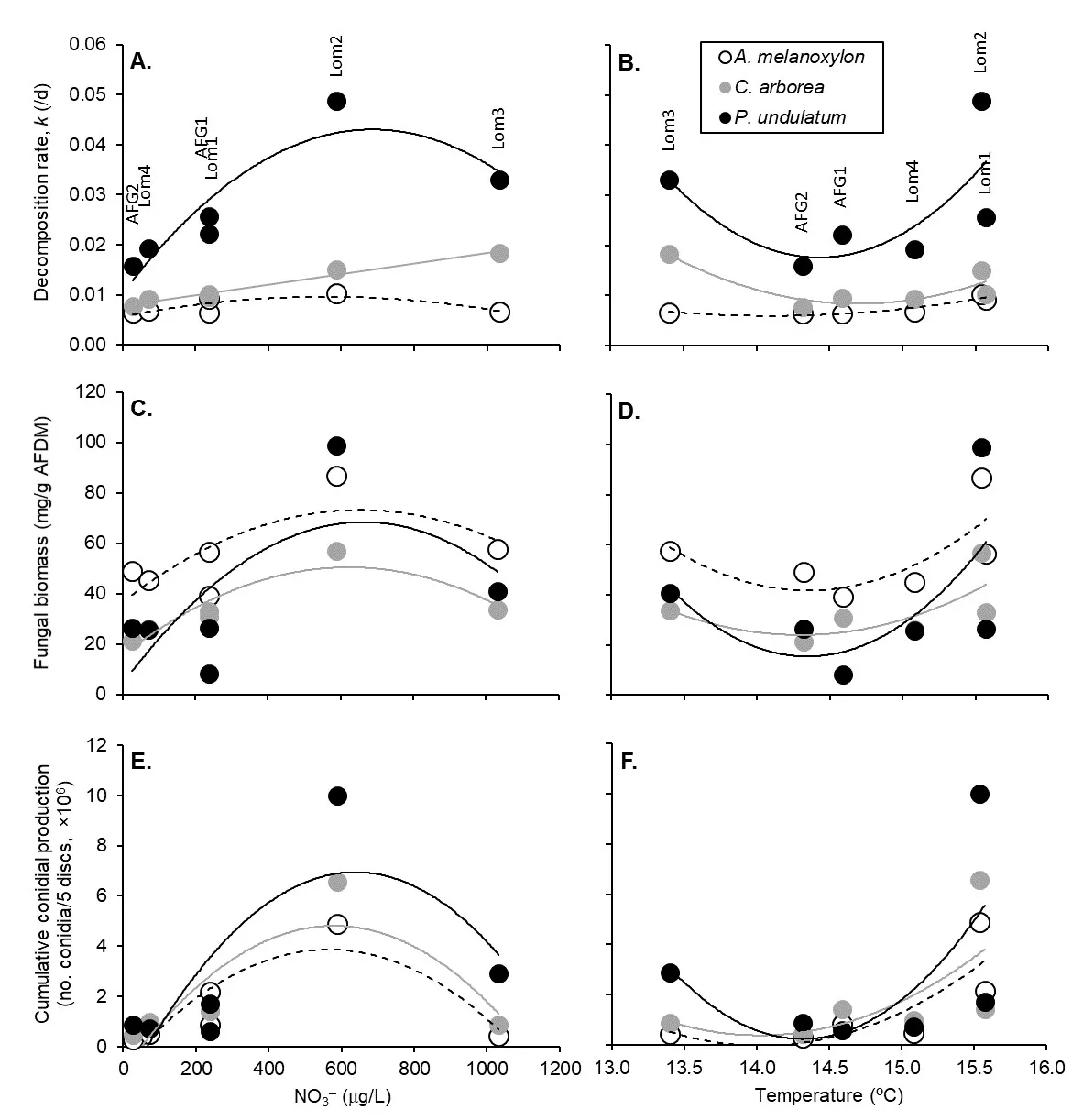



**Table:** 

Water variables (x-axis)	Litter species	Curve shape	Vertex x-axis	Vertex y-axis	R<sup>2</sup>
<b>A.</b>					
NO<sub>3</sub><sup>-</sup> (μg/L)	<i>A. melanoxylon</i>	Hump-shaped	500	0.0082	0.65
	<i>C. arborea</i>	Linear	-	-	0.97
	<i>P. undulatum</i>	Hump-shaped	714	0.046	0.84
Temperature (°C)	<i>A. melanoxylon</i>	U-shaped	14	0.0081	0.88
	<i>C. arborea</i>	U-shaped	14.7	0.0047	0.79
	<i>P. undulatum</i>	U-shaped	14.4	0.0259	0.53
					
<b>B.</b>					
NO<sub>3</sub><sup>-</sup> (μg/L)	<i>A. melanoxylon</i>	Hump-shaped	654	73	0.52
	<i>C. arborea</i>	Hump-shaped	623	50	0.85
	<i>P. undulatum</i>	Hump-shaped	663	68	0.45
Temperature (°C)	<i>A. melanoxylon</i>	U-shaped	14.4	42	0.55
	<i>C. arborea</i>	U-shaped	14.3	24	0.49
	<i>P. undulatum</i>	U-shaped	14.3	15	0.39
					
<b>C.</b>					
NO<sub>3</sub><sup>-</sup> (μg/L)	<i>A. melanoxylon</i>	Hump-shaped	565	3859639	0.77
	<i>C. arborea</i>	Hump-shaped	580	4805520	0.69
	<i>P. undulatum</i>	Hump-shaped	639	6941649	0.61
Temperature (°C)	<i>A. melanoxylon</i>	U-shaped	14	-28219	0.65
	<i>C. arborea</i>	U-shaped	14	384022	0.41
	<i>P. undulatum</i>	U-shaped	14.3	241257	0.39

## Description

Leaf litter decomposition is a key ecosystem process in forest streams, where litter inputs from the riparian vegetation are the main source of carbon and nutrients for aquatic food webs (Wallace et al., 1997). In island streams, where invertebrate shredders are generally rare (Benstead et al., 2009; MacKenzie et al., 2013; Raposeiro et al., 2014), litter decomposition is mostly driven by microbial decomposers, aquatic hyphomycetes in particular (Benstead et al., 2009; Larned 2000; MacKenzie et al., 2013; Raposeiro et al., 2014; Ferreira et al., 2016). Microbial decomposers are highly responsive to water temperature, with increases in temperature within organisms’ thermal ranges stimulating metabolic rates, and consequently the mineralization of litter carbon into CO _2_ through respiration (Ferreira and Chauvet 2011; Pérez et al., 2023). Microbial decomposers are also highly sensitive to nutrient availability, with increases in dissolved nutrient concentrations stimulating the use of litter as a source of carbon (Gulis and Suberkorpp 2003; Ferreira and Chauvet 2011). Consequently, increases in water temperature and nutrient availability generally translate into accelerated microbial-driven litter decomposition rates (Ferreira and Chauvet 2011; Fernandes et al., 2014). However, in island streams, variation in water temperature and nutrient concentrations, which may occur over a short distance due to steep changes in elevation and geology, do not necessary occur in the same direction, which complicates predictions of litter decomposition rates.

In this study, we incubated leaf litter of three woody species [ *Acacia melanoxylon* (R. Br.), *Clethra arborea* (Aiton), and *Pittosporum undulatum* (Vent.)] in 0.5-mm mesh bags, for up to 56 days (with four sampling dates), in six streams on São Miguel island, North Atlantic Ocean, to assess microbial-driven litter decomposition rates, fungal biomass, and conidia production by aquatic hyphomycetes. The temporal dynamics of the biotic variables over the incubation period can be found in Ferreira et al. (2016), while here we report on the relationships between the biotic variables and water nitrate concentration and temperature to assess the interaction between these two abiotic variables in moderating biotic responses under natural settings.

The relationship between biotic variables and nitrate concentration generally followed a hump-shaped curve, with maximum decomposition rates (across species) of 0.0082 – 0.0460/d estimated at 500 – 714 μg NO _3_^–^ /L, maximum fungal biomass of 50 – 73 mg/g AFDM estimated at 623 – 663 μg NO _3_^–^ /L, and maximum cumulative conidial production of 3.8 million – 6.9 million conidia/5 leaf discs estimated at 565 – 639 μg NO _3_^–^ /L ( Figure 1, Table 1). The relationship between decomposition rates and nitrate concentration was linear for *C. arborea* ( Figure 1, Table 1). The relationship between biotic variables and water temperature followed a U-shaped curve, with minimum decomposition rates (across species) of 0.0047 – 0.0259/d estimated at 14.0 – 14.7 ºC, minimum fungal biomass of 15 – 42 mg/g AFDM estimated at 14.3 – 14.4 ºC, and minimum cumulative conidial production of –28219 – 384022 conidia/5 leaf discs estimated at 14.0 – 14.3 ºC ( Figure 1, Table 1).

Litter decomposition and associated microbial activities were stimulated when nutrient availability and water temperature increased simultaneously across the group of streams including Lom1, Lom2, Lom4, AFG1, and AFG2 (Figure 1), as anticipated (Ferreira and Chauvet 2011; Fernandes et al., 2014). This stimulation was more evident for the fast-decomposing *P. undulatum* litter than for the other two species. This suggests that litter is more sensitive to increases in nutrient availability and temperature at more advanced stages of decomposition, which can be due to better microbial colonization of the litter and to the litter increased recalcitrance and thus higher dependence of decomposers on dissolved nutrients. Consistent with this interpretation, other studies found stronger differences in litter mass remaining across streams mostly at later sampling dates (Gulis and Suberkropp 2003; Ferreira et al., 2016, 2021).

However, when an increase in nutrient concentrations was accompanied by a decrease in water temperature (stream Lom3), litter decomposition and associated microbial activities were lower compared with what was expected from the increase in nutrient concentrations alone (see hump-shaped relationships) and higher when compared with what was expected from the decrease in water temperature alone (see U-shaped relationships) (Figure 1). This suggests that water temperature was the limiting factor for maximum litter decomposition rates and associated microbial activities in this stream. The role of temperature as a limiting factor was observed when comparing temperatures that cannot be considered extreme (13.4 – 15.5 ºC) and are within a short interval (2.1 ºC), suggesting that even small changes in temperature may have strong effects on decomposer activity and litter decomposition. Contrarily, higher nutrient availability may alleviate the effect of low water temperature as an increase in nutrient availability stimulated litter decomposition and associated microbial activities at lower temperature, although generally not at levels observed when temperature was higher. This agrees with Fernandes et al. (2014), who showed that higher nitrogen concentrations are needed to achieve maximum microbial activities at lower temperature.

Although the relationship between biotic variables and water temperature was described by a U-shaped curve, there was high variability at the highest temperatures with stream Lom2 showing higher litter decomposition rates (1.1 – 2.3-fold), fungal biomass (1.5 – 4.6-fold), and cumulative conidial production (1.9 – 5.9-fold) than stream Lom1 (Figure 1). This variation in biotic variables between both streams with similar water temperature (mean ± SE: 15.6 ± 0.1 ºC in Lom1 and 15.5 ± 0.1 ºC in Lom2) can be attributed to differences in dissolved nutrient concentration, which was 2.5-fold higher in stream Lom2 (588 ± 19 mg/L) than in stream Lom1 (238 ± 24 mg/L). This suggests that lower nutrient concentration was the limiting factor for maximum litter decomposition rates and associated microbial activities in stream Lom1.

Taken together, the increase in biotic variables with simultaneous increase in water nutrients and temperature, the limitation of biotic variables by lower water temperature at stream Lom3, and the limitation of biotic variables by low nitrate concentration at stream Lom1 suggest that decomposer activity and litter decomposition are reduced if one factor is unsuitable despite other factors being adequate, in agreement with the “concept of thresholds” (Prescott 2010). Given the small number of streams included, this conclusion is preliminary and requires further investigation.

## Methods

Leaf litter incubation was carried out in six small streams in the central massif of São Miguel island, Azores archipelago, North Atlantic Ocean. Four streams were tributaries of Ribeira Grande stream in Lombadas valley (Lom1 – Lom4) and two streams were tributaries of Fogo Lagoon (AFG1 and AFG2). Streams had similar geomorphology, were not visibly affected by direct human activities, were slightly alkaline (pH 7.7 – 8.1) and well oxygenated (8.4 – 9.1 mg O _2_ /L), and varied in water temperature (13.4 – 15.6 ºC) and nutrient concentrations (28 – 1033 μg NO _3_^–^ /L and 30 – 175 μg PO _4_^3–^ /L). Water temperature was recorded hourly over the 56-days incubation period during Spring and Summer 2014 using data loggers (Hobo Pendant UA-001-08, Onset Computer Corp., Massachusetts, USA) and hourly values were used to estimate daily means, which were averaged for each stream. On five occasions, stream water was filtered (fiberglass filters, 47-mm diameter, GF/C, Whatman, GE Healthcare Europe GmbH, Little Chalfont, UK) and used to determine nitrate concentration (ion chromatography; APHA 1995). A detailed description of the study area and streams is provided in Ferreira et al. (2016).

Air-dried leaves of three evergreen woody species commonly found in the riparian vegetation of Azorean streams were used to provide a gradient of litter palatability, in increasing order: *Acacia melanoxylon* (R. Br.) < *Clethra arborea* (Aiton) < *Pittosporum undulatum* (Vent.) (Ferreira et al., 2016). Leaves were weighed (2.90 – 3.10 g) and enclosed in fine-mesh bags (10 × 15 cm, 0.5-mm mesh), which prevent invertebrate access and where decomposition is mostly driven by microbial decomposers. Twelve litter bags per species were deployed in each stream on 11 or 12 June, 2014. Three replicate litter bags were recovered from each stream after 7, 21, 35 and 56 days, and transported cold to the laboratory. In the laboratory, litter was rinsed with distilled water and two sets of five leaf discs were extracted with a cork borer (12-mm diameter): one set was frozen at – 18 ºC for later determination of fungal biomass (Gessner 2020) and one set was used fresh to induce conidia production by aquatic hyphomycetes (Bärlocher 2020). The bulk litter mass was used to determine litter mass remaining (Ferreira et al., 2016).

For determination of fungal biomass, five frozen leaf discs were lyophilized (LY3TE, Snijders Scientific, Tilburg, The Netherlands), weighed (Kern 870, Kern & Sohn GmbH, Balingen, Germany) to determine dry mass, and incubated in alkaline methanol (8 g KOH/L, 30 min at 80 ºC) to extract ergosterol. The extract was purified using solid phase extraction cartridges (Waters Sep-Pak® Vac RC tC18 cartridges; Waters Corp., Massachusetts, USA) and eluted with isopropanol. Ergosterol was quantified by high-performance liquid chromatography (Dionex DX-120, California, USA), using a Thermo Scientific Syncronis C18 column and a Thermo Universal Uniguard holder 4/4.6 mm ID3 + Syncronis C18 drop in guard pre-column (Thermo, Waltham, Massachusetts, USA); the mobile phase was 100% methanol, kept at 33 ºC and flowing at 1.4 mL/min. Ergosterol was detected by reading absorbance at 282 nm with a UV detector and absorbance was converted into ergosterol concentration using a standard curve of ergosterol in isopropanol. Ergosterol concentration was converted into fungal biomass considering 5.5 mg ergosterol/mg fungal dry mass (Gessner and Chauvet 1993) and fungal biomass was expressed as mg/g litter AFDM, averaged across sampling dates.

Conidia production by aquatic hyphomycetes was determined after incubating five fresh leaf discs in 25 mL of filtered stream water (48 h at 13 ºC, 10 h light:14 h dark photoperiod, and 100 rpm). The conidia suspension was preserved with 2 mL of formalin and leaf discs were processed as described below for determination of discs ash-free dry mass. Suspensions were homogenized with 150 mL Triton X-100 and a magnetic stirring bar, and aliquots were filtered (nitrocellulose filters, 25-mm diameter, 5-mm pore size; Sartorius Stedim Biotech GmhH, Göttingen, Germany). Filters were stained with trypan blue in lactic acid and mounted on a slide. Conidia were counted at 320× magnification (DM1000 microscope, Leica, Wetzlar, Germany). Rates of conidia production were expressed as no. conidia/mg leaf AFDM/day, and cumulative (total) conidial production over the incubation period was estimated as the sum of daily values, which were derived by linear interpolation of the values of the adjacent sampling dates, and expressed as no. conidia/5 leaf discs.

For determination of litter mass remaining, litter (after extraction of leaf discs) was oven-dried (70 ºC, 48 h) and weighed (Kern 870, Kern & Sohn GmbH, Balingen, Germany) to assess dry mass (DM) remaining. DM remaining was ignited (500 ºC, 8 h) and weighed to assess ash mass. Ash-free dry mass (AFDM) remaining was estimated as the difference between DM and ash mass (after accounting for the extracted discs), and the fraction of AFDM remaining was estimated as the ratio between AFDM remaining and initial AFDM. Initial AFDM of samples was estimated as the product of initial air-dry mass by a conversion factor derived from extra sets of five fine-mesh bags per species. These extra bags, were prepared as the samples, taken to the field on day 0, immersed in water for ~ 10 min, and returned to the laboratory for determination of DM and AFDM as described above. The conversion factor was estimated as the ratio between initial AFDM and initial air-dry mass. Fraction AFDM remaining across the incubation times was used to estimate the overall exponential decomposition rate ( *k* , /days) for each species and stream as the slope of a linear regression between ln(fraction of AFDM remaining) and time (days).

The relationships between biological variables (microbial-driven litter decomposition rates, average fungal biomass, and cumulative conidial production) of the three leaf litter species and abiotic variables [nitrate concentration (average across sampling dates) and water temperature (average across 56 days)] in six streams were analyzed by fitting the data to a quadratic function. The relationship between litter decomposition rates and nitrate concentration for *C.arborea* was fitted to a linear model. Models were derived using Statistica 7 software (StatSoft Inc., Tulsa, Oklahoma, USA).
